# Supplementary material for: Multiple carbon cycle mechanisms associated with the glaciation of Marine Isotope Stage 4
Source: Nat Commun. 2022 Sep 16;13:5443. doi: 10.1038/s41467-022-33166-3 (PMC9481522; doi:10.1038/s41467-022-33166-3)
Supplement: Supplementary file 1 — Supplementary Information [file 41467_2022_33166_MOESM1_ESM.pdf]

SUPPLEMENT TO MULTIPLE CARBON CYCLE MECHANISMS ASSOCIATED WITH THE GLACIATION OF MARINE ISOTOPE STAGE 4

Carbon Cycle Model Experiments

As a heuristic tool for understanding the processes that control atmospheric CO_2, it is instructive to perturb models of the carbon cycle and examine the results on a cross-plot of $\delta^{13}C$-CO_2 and CO_2 concentration [1, 2, 3, 4]. Similar cross plots were first presented by Keeling [5] to interpret simple two end-member mixing of air masses. In the case of a perturbation to the carbon cycle where two-component mixing is complicated by interactions between the atmosphere, ocean, and terrestrial carbon reservoirs, a model must be used to quantify the short-term equilibration with the surface ocean and the longer-term equilibration with marine $CaCO_3$ sediments. For the purposes of interpreting ice core data, it is useful to plot the modeled changes in $\delta^{13}C$-CO_2 and CO_2 concentration after a perturbation and compare the slopes in the cross-plot to the slopes resolved by the data. We note that this way of interpreting the data is independent of the timing of CO_2 and $\delta^{13}C$-CO_2 changes, except for changes in the size of the terrestrial biosphere, in which case atmospheric $\delta^{13}C$-CO_2 becomes more negative the faster the release of CO_2 [3, 6].

The OSU 14-box model was previously used to simulate perturbations to the carbon cycle that would have been active during the last deglaciation, including changes in marine biological productivity, increased sea surface temperatures, increased Southern Ocean gas exchange rates, releases of terrestrial carbon, changes in circumpolar deep-water formation, and carbonate compensation in response to carbon transfer across the atmosphere-ocean interface[3]. We compiled results from the OSU box model and tested whether the perturbations, when run in the opposite direction (e.g., cooling SST instead of warming), resulted in linear changes in $\delta^{13}C$-CO_2 and CO_2 with respect to the original perturbations (Supplementary Figure 1). Generally, this was the case with an exception being Southern Ocean air sea gas exchange rate, which, when reduced, results in $\delta^{13}C$-CO_2 and CO_2 changes that plot slightly less vertically on the cross-plot (Supplementary Figure 1). We also tested the response of the atmosphere to the magnitude of perturbations and found that, generally, the $\delta^{13}C$-CO_2 and CO_2 changes scale linearly within the uncertainty of the data (Supplementary Figure 1).

Results of cooling SST were taken from Shackleton et al. [7], in which mean ocean temperature (MOT) and δD data from Antarctic ice cores were used to constrain the evolution of ocean temperature between MIS 5e-4. The full magnitude of the MOT change from MIS 5e-4 is approximately 4x that which occurred across the MIS 5a-4 transition, with the majority of the cooling occurring between MIS 5e-5a. The result illustrates the linearity of $\delta^{13}C$-CO_2 and CO_2 with respect to the magnitude of the SST changes (Supplementary Figure 1). The total effect of ocean solubility change due to -0.9 ˚C ocean cooling across MIS 5a-4 transition is 9 ppm decrease in CO_2 [7] and 0.13 ‰ decrease in $\delta^{13}C$-CO_2. The change in $\delta^{13}C$-CO_2 due to ocean cooling alone is much too small to explain the dramatic decrease in $\delta^{13}C$-CO_2 (interval II in Figure 1B, see main text).

In addition, we tested extension/ reduction of sea ice in the high-latitude boxes of the model. Sea ice extension causes a reduction in air-sea gas exchange and biological productivity in the box model (and the reverse is true with decreased sea ice extent causing increased air-sea gas exchange and increased biological productivity). The resulting effects on $\delta^{13}C$-CO_2 and CO_2 of sea ice changes vary substantially for Antarctic versus North Atlantic sea ice, the simplest rationale being that the Southern Ocean is a source of CO_2 in the model while the North Atlantic is a sink. Reducing (or increasing) sea ice extent over both high-latitude boxes may therefore have a canceling effect on CO_2 concentration, but with net negative (or positive) changes in $\delta^{13}C$-CO_2 (Supplementary Figure 1). Simultaneous sea ice fluctuations in the northern and southern high latitudes are therefore a possible explanation for the large changes in $\delta^{13}C$-CO_2 that occurred with nearly zero change in CO_2 concentration during MIS 4 (interval III, see main text). The light-blue region representing sea ice covers a wide swath of the cross-plot to represent potential combinations of North Atlantic and Antarctic sea ice change, with the boundaries representing perturbations to only the south or the north. As observed by another study [1], a decrease in Antarctic sea ice results in a significant decrease in $\delta^{13}C$-CO_2 because it activates a source of light carbon to the atmosphere. A very sharp decrease in $\delta^{13}C$-CO_2 can also result from increasing the Southern Ocean gas exchange parameter (purple dashed lines in Supplementary Figure 1), which could result from a change in the strength or position of the southern hemisphere westerlies.

The data spanning 74-59 ka require processes that plot steeply on the cross-plot, and it is for this reason the large changes in $\delta^{13}C$-CO_2 resulting from sea ice and Southern Ocean gas exchange perturbations form an important basis for the hypotheses discussed in the main text of this manuscript. We note that a similar result was obtained with the BICYCLE box model [1]. The LOVECLIM model of intermediate complexity also demonstrated large change in $\delta^{13}C$-CO_2 per change in CO_2 for transient deglacial experiments [8] and idealized experiments [9] in which the southern hemisphere westerlies affected air-sea gas exchange rates. We advocate for more extensive testing of these processes with complex models.

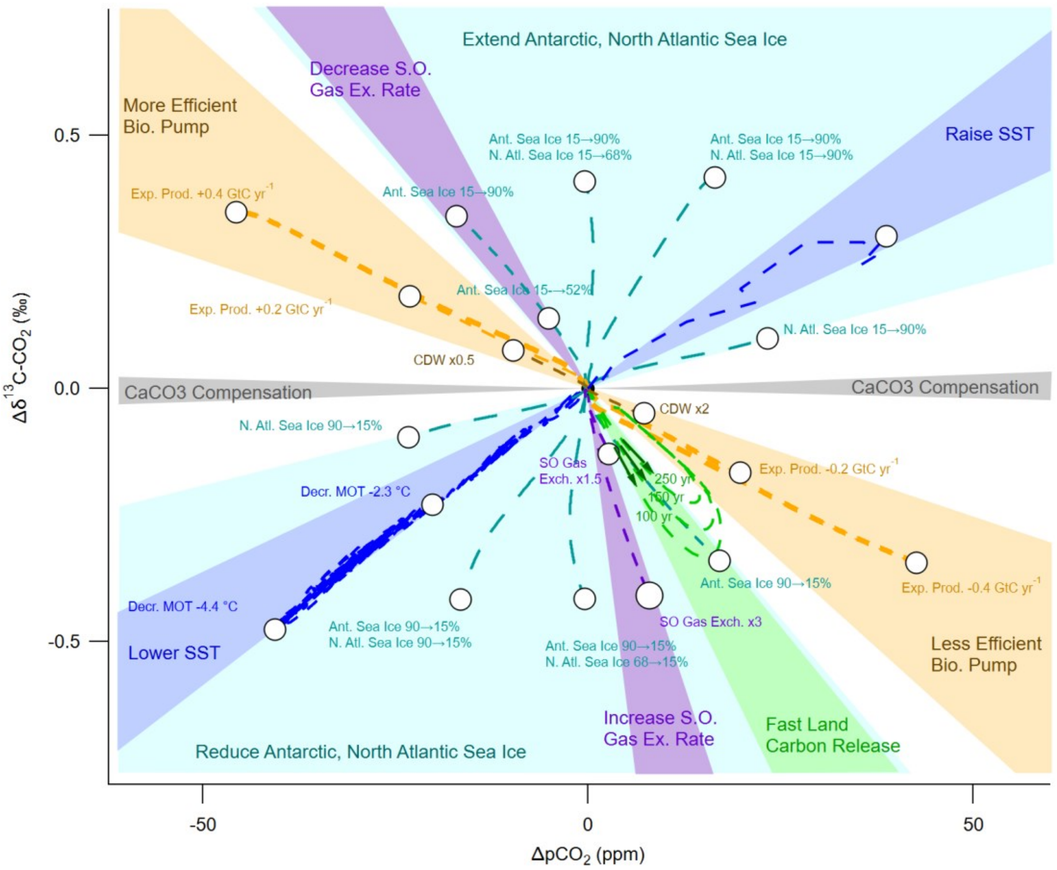

Supplementary Figure 1 – Change in atmospheric CO_2 and $\delta^{13}C$-CO_2 for perturbations to the carbon cycle in the OSU box model. We took the colored regions from cross-plots in the study of Bauska et al. 2016 [3], which bound the inter- and intra-model variability explored by Bauska 2016 for the last deglaciation, and extrapolated the regions backwards to represent perturbations that were likely active during the last glaciation (e.g., cooling SST versus warming). We plotted results from perturbing biological productivity, circumpolar deep water (CDW) formation rate, sea surface temperature (SST) cooling, changes in Southern Ocean air-sea gas exchange rate, land carbon release, and sea ice changes. The results are plotted as net change in $\delta^{13}C$-CO_2 and CO_2 relative to the origin. The circle markers represent atmospheric $\delta^{13}C$-CO_2 and CO_2 when the atmosphere has reached equilibrium, and the dashed lines represent the path traced in time with the origin representing t=0. An important point is that the changes in $\delta^{13}C$-CO_2 and CO_2 are linear within the uncertainties with respect to time except for fast land carbon release, which shows a steeper decrease in atmospheric $\delta^{13}C$-CO_2 for faster perturbations. The three green arrows trace 50 GtC land carbon releases over timescales of 100, 150, and 250 years, with gradual relaxation toward the origin as the released carbon equilibrates with the ocean. We did not plot a shaded region to represent carbon uptake by the terrestrial biosphere because we do not think this mechanism is relevant to the time period in our study. It would appear as the reflection of the green fast land carbon release shading and overlap significantly with the purple region representing decreased Southern Ocean gas exchange rate.

We compiled results of experiments conducted with four different carbon cycle models of varying complexity (Supplementary Figure 2) to see if, despite differences in experimental design and boundary conditions, different models achieved similar results in the cross-plot for similar

perturbations. The models were the Box Model of the Isotopic Carbon Cycle (BICYCLE) [1, 4], the Bern 3d Earth System Model [10, 11], the University of Victoria Earth System Climate Model (UVic ESCM) [12], and the isotope-enabled LOVECLIM earth system model of intermediate complexity.

The UVic ESCM was previously used to simulate changes in the strength of the Atlantic Meridional Overturning Circulation (AMOC) [13] (results from supplementary of Bauska et al. 2016 [3]), and we additionally used UVic to simulate increased productivity by marine phytoplankton.

The BICYCLE box model was previously used to test AMOC variations due to changes in NADW formation, as well as increased productivity, changes in sea surface temperatures, land carbon release, and reductions in North Atlantic and Antarctic sea ice [1, 4]. Results of Bicycle experiments are from Eggleston et al. 2016 [2] and Kohler et al. 2006 [1].

The LOVECLIM model of intermediate complexity was used to test the effect of ocean circulation changes on $\delta^{13}C$-CO_2 and CO_2 in idealized experiments [9]. The LOVECLIM was also used to simulate a transient deglacial scenario in which intensified southern hemisphere westerlies windstress achieved a fast (within 100 years), decrease of -0.2‰ in $\delta^{13}C$-CO_2 at 16.2 ka [8] associated with an 8 ppm rise in CO_2 concentration.

We also included results from the Bern 3d Earth System Model of intermediate complexity including changes in Southern Ocean upwelling, AMOC variations, and iron limitation [10, 11] (results from supplementary of Bauska et al. 2016 [3]).

While generally the various model results agree in terms of the expected change in $\delta^{13}C$-CO_2 and CO_2, there are some results that fall outside of the expected region. This is not surprising given the differences in model geometry, boundary conditions, and experimental design. Importantly, changes in the efficiency of the ocean biological pump (either due to changes in circulation or productivity) and sea surface temperatures tend to result in $\delta^{13}C$-CO_2 and CO_2 changes with shallower slopes in the cross-plot than fast land carbon transfers, changes in Southern Ocean air-sea gas exchange rate, and certain sea ice changes (an exception being changes in North Atlantic sea ice coverage). For the purposes of presenting the high-resolution $\delta^{13}C$-CO_2 and CO_2 data from 74-59 ka, the compilation of model results forms a useful heuristic for developing hypotheses about what mechanisms forced CO_2 to change at different intervals. A weakness, however, is that the analysis is largely limited to qualitative assessment of what processes drove the changes.

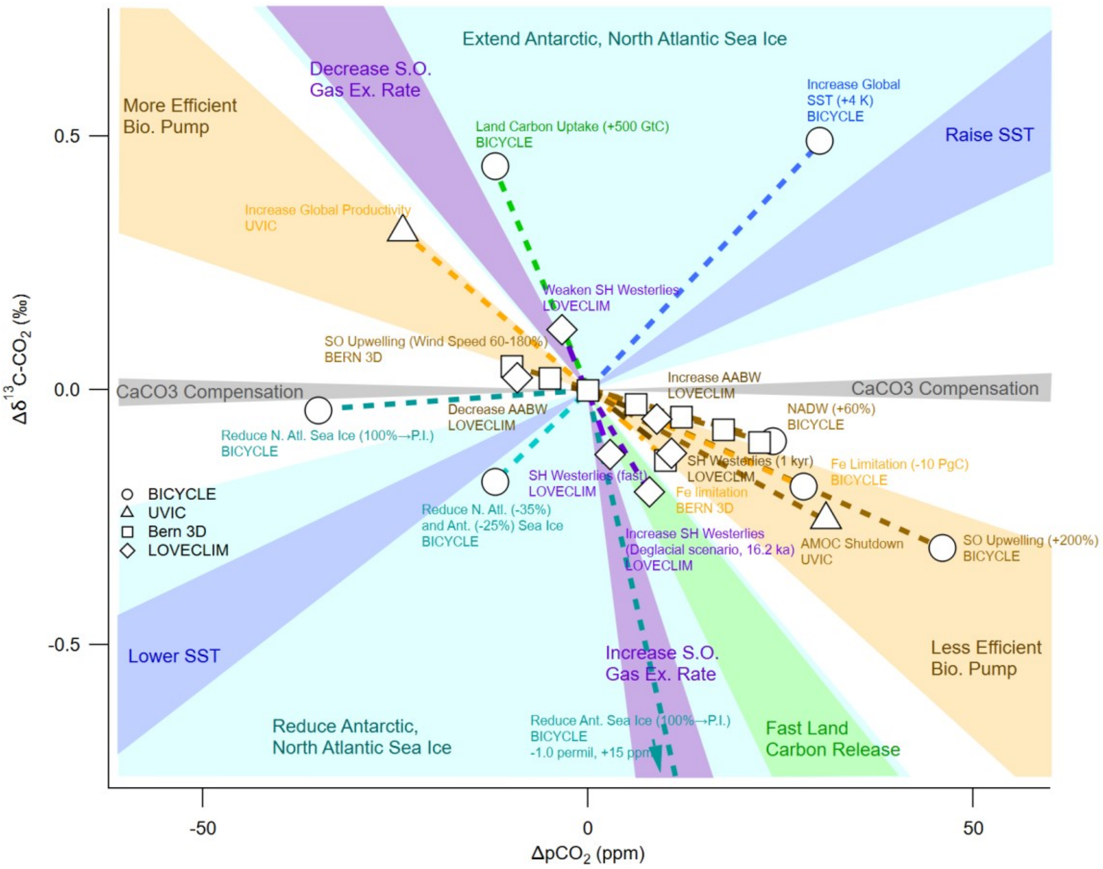

Supplementary Figure 2 – Change in atmospheric CO_2 and δ^{13}C-CO_2 from perturbations to the carbon cycle in various biogeochemical models. The model results come from BICYCLE (circles), UVic (triangles), Bern3d (squares), LOVECLIM (diamonds), and the OSU box model (shaded regions, see Supplementary Figure 1). The markers indicate the magnitude of change in CO_2 and δ^{13}C-CO_2 for a particular perturbation. The shaded regions represent our grouping of perturbations: biological pump (yellow), sea ice (light blue), land carbon release (green), Southern Ocean gas exchange (purple), sea surface temperature (blue), alkalinity (gray).

Supplementary Table 1 – Description of model experiments compiled in Supplementary Figure 1 and Supplementary Figure 2. For the experiments conducted in this study and by Bauska et al. 2016 [3], the difference in $\delta^{13}C$-CO_2 and CO_2 was computed from the starting and ending values after the perturbation had equilibrated between all reservoirs. Land carbon transfers are an exception where the time-dependent transient change in $\delta^{13}C$-CO_2 and CO_2 differed depending on the size and timing of the carbon pulse. For results from Eggleston et al. 2016 [2] (originally from Kohler et al. 2010[4] and Kohler et al. 2006[1]), the change in $\delta^{13}C$-CO_2 and CO_2 was computed relative to a control run.

	Model	Reference	Perturbation
Sea Ice	OSU Box	This study	Extend Ant. SI 15→90%
	OSU Box	This study	Extend Ant. SI 15→90%, Extend N. Atl. SI 15→68%
	OSU Box	This study	Extend N. Atl. SI 15→90%
	OSU Box	This study	Reduce Ant. SI 90→15%
	OSU Box	This study	Reduce Ant. SI 90→15%, Reduce N. Atl. SI 68→15%
	OSU Box	This study	Reduce N. Atl. SI 90→15%
	BICYCLE	Kohler et al. 2006	Reduce N. Atl. SI 100%→Preindustrial
	BICYCLE	Kohler et al. 2010	Reduce Southern Ocean (-35 %) and North Atlantic (-25 %) sea ice coverage
	BICYCLE	Kohler et al. 2006	Reduce Ant. SI 100%→Preindustrial
Sea Surface Temperature	OSU Box	Shackleton et al. 2021	Lower global average sea surface temperature -4.4 ˚C, scale SSTs to EDC δD
	OSU Box	Bauska et al. 2016	Raise sea surface temperatures (deglacial simulation)
	BICYCLE	Kohler et al. 2010	Raise average global sea surface temperatures +4 K
$CaCO_3$ Compensation	OSU Box	Bauska et al. 2016	Response to carbon transfer across atmosphere-ocean interface
Biological Pump Efficiency (Productivity or Circulation)	Bern 3D	Tschumi et al. 2011	Increase Southern Ocean upwelling, varied wind speed 60→180 % (AMOC on)
	BICYCLE	Kohler et al. 2010	Strengthen NADW +60%
	BICYCLE	Kohler et al. 2010	Increase Southern Ocean upwelling +200%
	UVic	Schmittner and Lund 2015	AMOC Shutdown
	UVic	This study	Increase global productivity by raising phytoplankton growth rates
	Bern 3D	Menviel et al. 2012	Increase productivity by enhancing iron limitation
	LOVECLIM	Menviel et al. 2015	Enhance southern hemisphere westerlies (1000 yr)
	LOVECLIM	Menviel et al. 2015	Weaken AABW formation
	LOVECLIM	Menviel et al. 2015	Strengthen AABW formation
	OSU Box	This study	Increase Southern Ocean export by decreasing restoring phosphate
	OSU Box	This study, Bauska et al. 2016	Decrease Southern Ocean export by increasing restoring phosphate
	OSU Box	Bauska et al. 2016, This study	Increase CDW formation rate +100%
	OSU Box	This study	Decrease CDW formation rate -50%
	BICYCLE	Kohler et al. 2010	Decrease productivity by enhancing iron limitation -10 PgC
S.O. Gas Exchange	LOVECLIM	Menviel et al. 2015	Strengthen southern hemisphere westerlies with air-sea gas exchange (fast, before equilibration)
	LOVECLIM	Menviel et al. 2015	Weaken southern hemisphere westerlies with air-sea gas exchange
	LOVECLIM	Menviel et al. 2018	Deglacial scenario with southern hemisphere westerlies windstress, 16.2 ka
	OSU Box	Bauska et al. 2016, This study	Increase Southern Ocean gas exchange parameter +50%, + 300%
Terrestrial Biosphere	OSU Box	Bauska et al. 2016, this study	Fast land carbon transfer (+/- 50 GtC) on 100, 150, 250 year timescale
	BICYCLE	Kohler et al. 2010	Grow terrestrial biosphere +500 PgC

Forward Modeling of $\delta^{13}C$-CO_2 and CO_2 74.0-59.5 ka

We used the OSU box model to conduct forward simulations of the carbon cycle changes proposed in the main text at each of the four intervals: the rise in CO_2 at DO 19, the MIS 5-4 CO_2 drop, $\delta^{13}C$-CO_2 changes during MIS 4 while CO_2 was stable, and the CO_2 rise during Heinrich Stadial 6. The forward simulations allow us to estimate the potential magnitude of forcing required to explain the data and demonstrate the challenges in reproducing the data at certain intervals.

CO_2 Increase and Isotopic Excursion at DO-19

The negative isotope excursion associated with DO-19 can only be explained by an input of very light CO_2 to the atmosphere. Simple models have suggested that such a pulse of carbon could come from a rapid release of terrestrial carbon, or a change in the air-sea gas exchange rate over the Southern Ocean [1, 3]. To our knowledge, the large effect of gas exchange on $\delta^{13}C$-CO_2 has primarily been documented in highly simplified box models (OSU box model, BICYCLE), which may represent a potential weakness of the result. Tests of the sensitivity of different models to Antarctic sea ice coverage (which affects the Southern Ocean air-sea gas exchange rate) showed large overestimations of the CO_2 drawdown caused by extending Antarctic sea ice [14, 15], probably due to the simplicity of the box model geometry. Atmospheric CO_2 was less sensitivity to sea ice in more complex models where air-sea gas exchange is more diffuse. This finding does not directly challenge our result because we invoke Southern Ocean gas exchange to explain large changes in *$\delta^{13}C$-CO_2* concurrent with *small* changes in CO_2, but we caution that the result could still be highly dependent on model architecture, as suggested by others [1]. We do note idealized simulations from the LOVECLIM earth system model of intermediate complexity show a fast and very negative trend in $\delta^{13}C$-CO_2 associated with strengthening the southern hemisphere westerlies in experiments where the wind stress affected the rate of air-sea gas exchange[9]. Simulations of the deglacial CO_2 rise also showed a very negative $\delta^{13}C$-CO_2 trend associated with southern hemisphere westerlies changes at 16.2 ka[8]. Both results agree broadly with our Southern Ocean air-sea gas exchange result, but we think future efforts with complex models should test whether large decreases in $\delta^{13}C$-CO_2 can in fact be achieved by altering Southern Ocean gas exchange rates.

We illustrate the DO-19 scenarios described in the text with forward simulations in the OSU box model. We simulated a rapid CO_2 rise at DO-19 if (1) all of the $\delta^{13}C$-CO_2 change came from S.O. gas exchange, (2) if all of the CO_2 change came from a fast release of land carbon, and (3) if sea surface temperature changes constrained by mean ocean temperature data contributed to the CO_2 rise due to decreased ocean solubility (Supplementary Figure 3). The model demonstrates that a 3.5x increase in the S.O. gas exchange rate achieves the same slope in the cross-plot space as the data, but still falls short in explaining the full magnitude of CO_2 and $\delta^{13}C$-CO_2 change observed at DO-19. We consider this a very hard push of the model, and further do not suspect shifting Westerlies to operate as abruptly in phase with Northern Hemisphere warming as the observed CO_2 rise. We therefore prefer a land carbon explanation. However, driving the model solely with a land carbon transfer of 95 GtC cannot achieve the negative isotope anomaly without overshooting CO_2 concentration (Supplementary Figure 3). An estimate of CO_2 rise due to sea surface temperature changes across DO-19 [7] constrained by ice core mean ocean temperature data shows that the + 0.26 ˚C mean ocean temperature anomaly may be associated with a positive $\delta^{13}C$-CO_2 anomaly of ~ +0.08 ‰ and a positive CO2 anomaly of ~ +7 ppm prior to the onset of DO-19. This may suggest the isotopically light CO_2 pulse at DO-19 is even more difficult to explain because the atmosphere would have already gained enriched CO_2 prior to the onset of DO-19, thus requiring an even greater negative change in $\delta^{13}C$-CO_2 at the event. On the other hand, if the ocean was cooling during DO-19, as suggested by the phasing of the mean ocean temperature data, this may help explain the negative excursion if combined with a land carbon release because it would lower $\delta^{13}C$-CO_2 while also providing a compensating sink for the CO_2 overshoot.

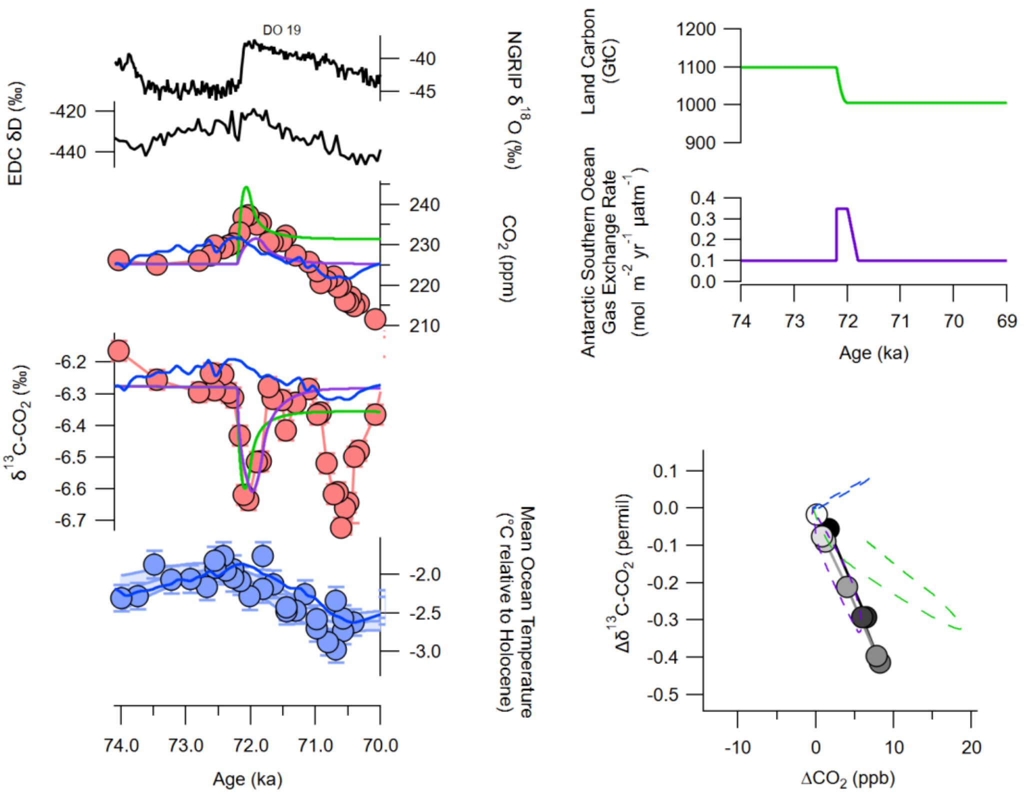

Supplementary Figure 3 – A forward model simulation of $\delta^{13}C$-CO_2 and CO_2 during DO-19. We demonstrate a 95 GtC release from the terrestrial biosphere (green line), a 3.5x increase in the Southern Ocean gas exchange rate (purple line), and the response to a +0.26 ˚C mean ocean temperature anomaly based on an estimate from Shackleton et al. 2021 (blue line) [7]. We prefer the land carbon scenario for reasons outlined in the text, but we note it is not possible to achieve the full magnitude of isotopic change without overshooting CO_2 concentration.

<u>CO_2 Decrease at the Onset of MIS 4</u>

The negative isotope anomaly at 70.5 ka is impossible to explain with only one carbon cycle process because the change in $\delta^{13}C$-CO_2 per change in CO_2 in the cross-plot is unlike any of the vectors attributed to individual perturbations (main text Figure 2d). We ruled out ocean cooling as a significant contributor between 71-69 ka [7]. We also suggest that $CaCO_3$ compensation played a minimal role in the CO_2 drawdown. The exact contribution of $CaCO_3$ compensation to the CO_2 drawdown is difficult to estimate because $CaCO_3$ compensation occurs over multi-thousand year timescales [16], and we do not have data prior to 74.0 ka to judge whether the marine $CaCO_3$ system was in a steady state or in a transient readjustment in response to events that occurred out of the interval captured by the ice core data. Assuming the marine $CaCO_3$ system was in equilibrium prior to the onset of DO-19, we used the OSU box model to estimate that $CaCO_3$ compensation could be responsible for an additional 2 ppm drawdown in CO_2 in response to a pulse of land carbon at the onset of DO-19 and the subsequent ocean CO_2 uptake (not shown). We do not have data to constrain carbon cycle changes prior to 74.0 ka (e.g., DO-20 and the transition to stadial conditions preceding DO-19), but conservatively, we estimate continued adjustment of the marine carbonate system with $CaCO_3$ sediments in response to an event at DO-20 could have conspired with the adjustments following DO-19 to cause a maximum 4 ppm reduction in CO_2 attributed to $CaCO_3$ compensation. This process would have occurred gradually over the course of the full MIS 5-4 transition and would do nothing to explain the large $\delta^{13}C$-CO_2 decrease and rebound centered at 70.5 ka.

We pose the hypothesis that a pulse of isotopically light carbon from an organic carbon reservoir caused the negative isotope anomaly. Such a pulse, however, causes too large of an increase in CO_2 and must be balanced by a compensating sink, such as enhancement of the biological pump combined with extension of Southern Ocean sea ice.

Forward modeling this scenario with the OSU box model demonstrates that the magnitudes of the perturbations required to reproduce the data are quite high, calling into question how plausible the scenario really is. In our best-case scenario (Supplementary Figure 4), there is a rapid transfer of ~260 GtC with $\delta^{13}C$ = -24.2 ‰ within 800 years. It is estimated from benthic $\delta^{13}C$ records that between 330-694 GtC was transferred from the terrestrial biosphere to the ocean between glacial versus interglacial times [17], but the timing of the transfer is not well known. While at the lower end of the estimated range, a carbon pulse of the magnitude in the model would represent a great portion of that total. The CO_2 increase from the land pulse with no other effects would raise atmospheric CO_2 by 40 ppm during the transient release, and 18 ppm after equilibration with the ocean. Smaller land carbon pulses are unable to achieve the same magnitude of isotope depletion. The biological pump must have rapidly increased efficiency to attenuate the accompanying CO_2 increase, evident in dust flux, ocean productivity, and ocean circulation proxy data (main text Figure 4), raising whole ocean DIC by about 20 µmol kg^{-1}. The compensating biological pump perturbation by itself would lower atmospheric CO_2 by 47 ppm, and the sea ice extension causes further sequestration of about 6.5 ppm. The sea ice is reasonable, however the biological pump term needed to compensate for the large land carbon pulse is either at the upper end of published estimates for total glacial-interglacial CO_2 change, or exceeding them [11, 18, 19, 20].

A potential alternative is that the negative isotope excursion occurred at the same time as D-O event 19.1, the warming reversal that occurred at the beginning of MIS 4. This would require errors in the GICC05A timescale, or the synchronization of AICC 2012 to it, of up to 2 ka at ~69.5 ka, which is within the published absolute uncertainties of the chronologies [21, 22]. If the negative isotope excursion aligned with the DO-19.1 reversal, then it is possible another flushing event drove isotopically light carbon out of the deep ocean when AMOC switched on, similar to what we proposed in the main text as a possible explanation for the preceding negative excursion at DO-19. In this case, the flushing must have caused changes in $\delta^{13}C$-CO_2 and CO_2 along a steeper vector than those produced by models that tested processes like upwelling and changes in circulation, which tend to plot in the yellow region of Supplementary Figure 2.

Overall, we conclude the negative isotope excursion at 70.5 ka is a difficult feature to explain with potentially important implications for land carbon-climate and ocean-climate feedbacks. We suggest that millennial-scale $\delta^{13}C$-CO_2 changes in MIS 5 and MIS 3 warrant further study and that the curious depletion trend during the MIS 5-4 transition might be an important clue for glacial-interglacial CO_2 dynamics.

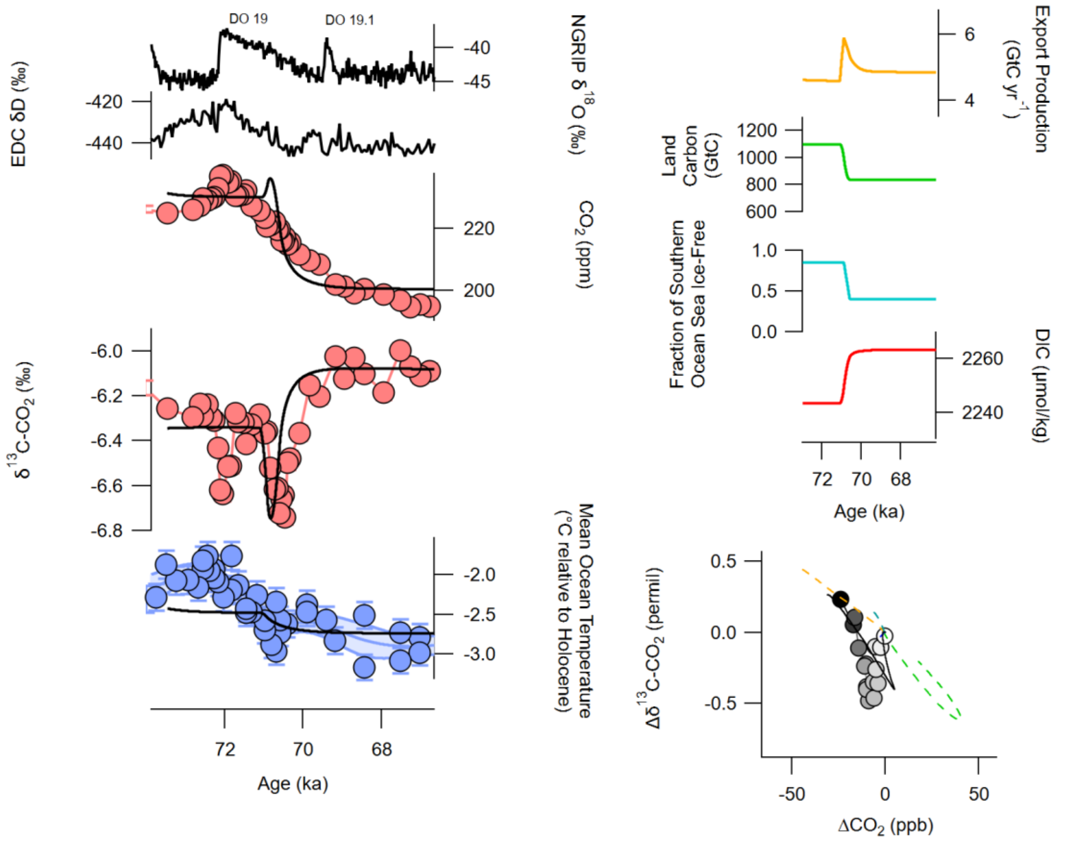

Supplementary Figure 4 - A forward model simulation of the CO_2 drop during the MIS 5-4 transition. It is impossible to achieve the large isotope anomaly without some pulse of isotopically light carbon. (Left) Our best scenario (black line) involves a combination a land carbon pulse and compensating sinks in the form of a rapid increase in export productivity from the biological pump and increase in Antarctic sea ice. The relatively small effect from ocean cooling was also included in the model and accounts for only a 3 ppm reduction in CO_2. (Right, Top) Carbon cycle perturbations used to drive the model. (Right, Bottom) Dashed lines in the cross-plot show effects of individual perturbations. The combined result (black curve) shows that the individual perturbations conspire additively to form a scenario that more closely resembles the data.

Low CO_2 and $\delta^{13}C$-CO_2 Variations During MIS 4

We attempted to simulate the large changes in $\delta^{13}C$-CO_2 associated with little to no change in CO_2 concentration that occurred during MIS 4 (interval III in main text Figure 1B). In the main text, we proposed a combination of mechanisms changed $\delta^{13}C$-CO_2 with a net canceling effect on CO_2 concentration. Recognizing that simultaneous changes in North Atlantic and Antarctic sea ice extent have such a canceling effect in box models [1], we forced the model with changes in sea ice extent in the high latitude surface boxes of the OSU model. Varying Antarctic sea ice extent between 0-95% coverage and North Atlantic sea ice between 10-25% coverage achieved changes in $\delta^{13}C$-CO_2 with only small changes in CO_2, but the resulting isotope variations were too small (Supplementary Figure 5). The reason is that the Southern Ocean biological pump, having been enhanced in the previous simulation to achieve the MIS 5-4 CO_2 drawdown, caused the $\delta^{13}C$ of dissolved inorganic carbon in the surface Southern Ocean to be more enriched. Hence, reductions in Antarctic sea ice during MIS 4 caused less reduction in atmospheric $\delta^{13}C$-CO_2 than they would have if the biological pump had been more limited, with $\delta^{13}C$ of surface Southern Ocean dissolved inorganic carbon more negative. For this reason, and also motivated by the MIS 4 dust variations observed in Antarctic ice cores[23, 24], we changed biological productivity in the Southern Ocean between 4.2-5.0 GtC yr^{-1}. We also prescribed a small mean ocean temperature change of -0.2 °C to be consistent with the cooling observed in the ice core mean ocean temperature data [7]. The fluctuations in biological pump efficiency gave more leverage to the Antarctic sea ice changes in terms of the effect on atmospheric $\delta^{13}C$-CO_2, better reproducing the MIS 4 data (Supplementary Figure 5). The forcings are admittedly ad hoc, but the results demonstrate

that it is possible to achieve $\delta^{13}C$-CO_2 changes on the order of ±0.2 ‰ with CO_2 changes on the order of only a few ppm. This being said, the large Antarctic sea ice changes are probably unrealistic given that MIS 4 was a period of relatively stable extreme cold, particular in the southern hemisphere. In light of this, we hope the main purpose of this model result is to highlight the difficulty in explaining the features observed in the MIS 4 data and stimulates future model work.

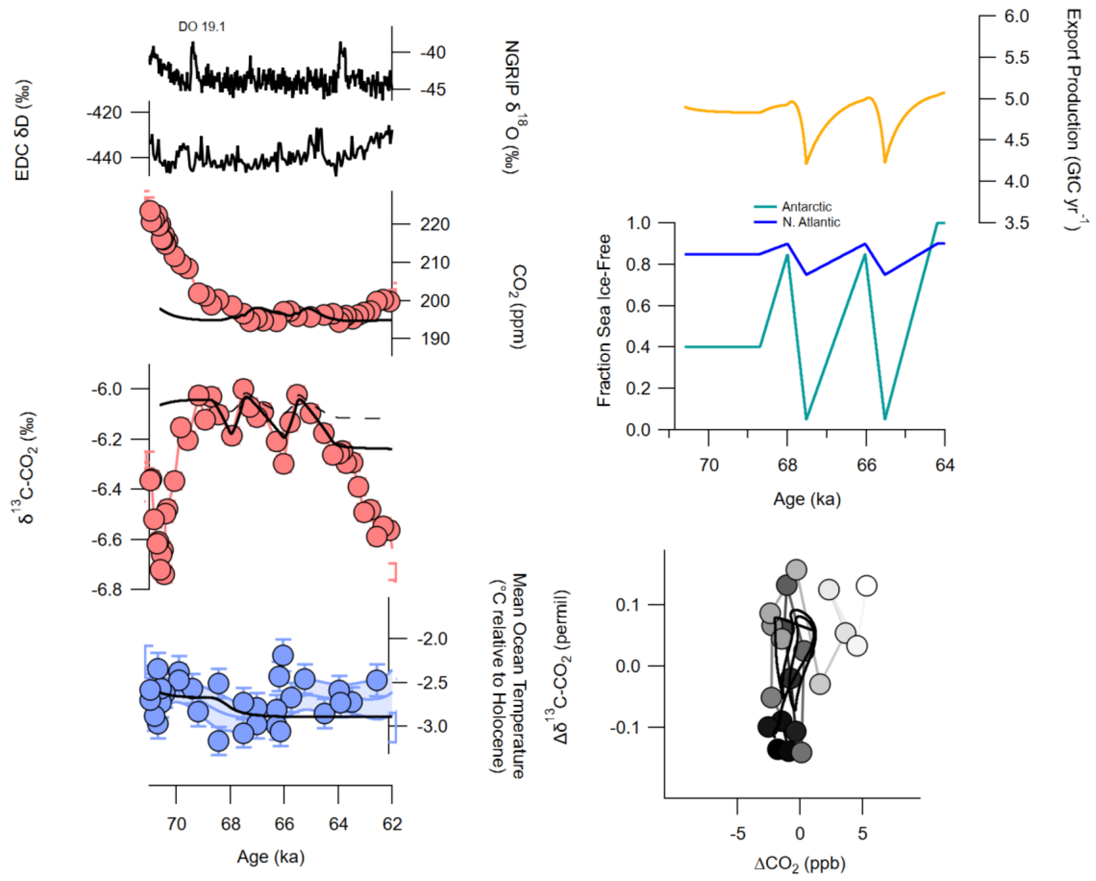

Supplementary Figure 5. A forward model simulation of the large $\delta^{13}C$-CO_2 fluctuations during MIS 4. Simultaneous changes in North Atlantic and Antarctic sea ice coverage have a canceling effect on atmospheric CO_2, but the effect on atmospheric $\delta^{13}C$-CO_2 is too small without changes in Southern Ocean biological productivity (the dashed line shows the $\delta^{13}C$-CO_2 resulting from the sea ice changes without biological pump changes). (Right, Top) Model forcings included changes in Southern Ocean biological productivity, and North Atlantic and Antarctic sea ice changes. (Right, Bottom) The model result (black curve) plots in a similar space on the cross-plot as the ice core data.

CO_2 Rise and $\delta^{13}C$-CO_2 decrease During the MIS 4-3 Transition

We simulated the CO_2 rise and large $\delta^{13}C$-CO_2 decrease across the MIS 4-3 transition (Heinrich Stadial-6) by combining the carbon cycle processes likely to have been active as proposed in the main text. The efficiency of the ocean biological pump was decreased by relaxing Southern Ocean biological productivity. Global export productivity decreased from 5.2-4.8 GtC yr^{-1}, decreasing whole ocean dissolved inorganic carbon by about 6.5 μmol kg^{-1}. Mean ocean temperature increased 1.2 ˚C in agreement with estimates by Shackleton et al. [7], which contributed about 10 ppm to the CO_2 rise and increased atmospheric $\delta^{13}C$-CO_2 by about 0.14 ‰. We did not decrease Antarctic sea ice because it was already decreased to 0 % coverage in the previous experiment in order to achieve the $\delta^{13}C$-CO_2 decrease from 65.5-64.0 ka. In order to get a large change in $\delta^{13}C$-CO_2 as observed in the data, we increased the Southern Ocean gas exchange parameter by four times. The combination of these forcings applied to the box model resulted in a $\delta^{13}C$-CO_2 decrease of -0.71 ‰, very near the full magnitude observed in the data, but the transition was accompanied by too much CO_2 increase, overshooting the measured CO_2 concentration by about 25 ppm.

We think the increase of the Southern Ocean gas exchange parameter may represent an extreme push of the model, but we note that the South Atlantic opal proxy for southern hemisphere westerlies shows the second largest increase in opal flux, only rivaled by the increase during the last deglaciation [25]. Thus, we think it is very likely that the southern hemisphere westerlies shifted south and/or strengthened during Heinrich Stadial-6, and that this helped drive isotopically depleted carbon out of the Southern Ocean and contributed to the very low atmospheric $\delta^{13}C$-CO_2.

The poor reproduction of the CO_2 data suggests that either our proposed scenario is flawed or that the box model insufficiently simulates the proposed processes. One possibility is that Antarctic sea ice decrease contributed to the MIS 4-3 CO_2 rise (which would provide an additional source of isotopically depleted CO_2 that would help decrease $\delta^{13}C$-CO_2). If so, a different process would need to be invoked to explain the isotope variations during MIS 4.

Overall, the modeling exercises presented in this supplementary material represent a preliminary attempt at quantifying the carbon cycle processes responsible for atmospheric CO_2 and $\delta^{13}C$-CO_2 during the period 74-59 ka. The simulations were intended to show the effect on CO_2 and $\delta^{13}C$-CO_2 if certain processes, or combinations of processes, were turning on/ off at different intervals as hypothesized in the main text. The forward simulations highlight where the data can be explained well with a simple box model, as well as intervals where the data are difficult to reproduce. We suggest that future work should focus on using $\delta^{13}C$-CO_2 to quantify changes in carbon cycle processes with special attention to what processes have a large influence on atmospheric $\delta^{13}C$-CO_2 relative to CO_2 concentration.

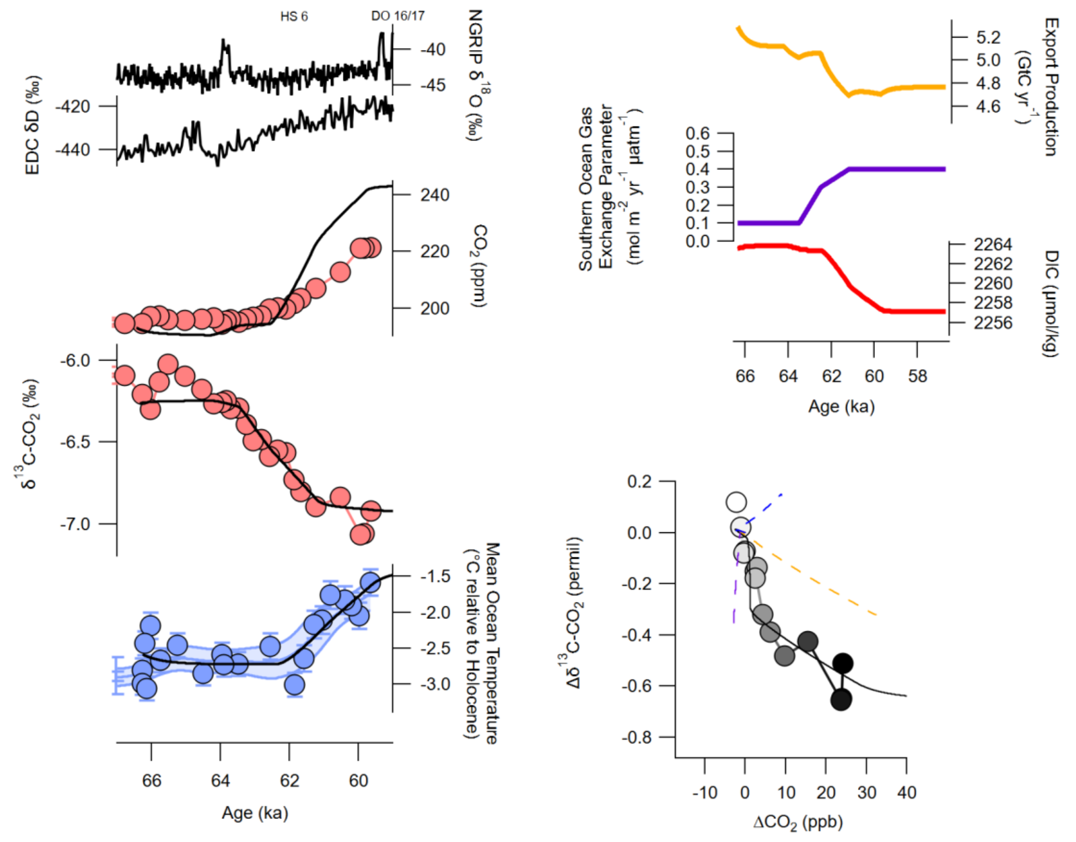

Supplementary Figure 6. A forward model simulation of the CO_2 rise and large $\delta^{13}C$-CO_2 decrease across the MIS 4-3 transition. Decreasing the efficiency of the ocean biological pump does not reduce atmospheric $\delta^{13}C$-CO_2 enough to reproduce the data. A large decrease in $\delta^{13}C$-CO_2 was achieved by increasing the Southern Ocean gas exchange parameter. Mean ocean temperature change is estimated to have increased CO_2 and $\delta^{13}C$-CO_2, opposing the trend seen in the isotope data and therefore representing a process that was masked by other carbon cycle processes active at the time. The overshoot in simulated CO_2 represents a weakness of the scenario, or the model, and highlights a target for future modeling efforts.

REFERENCES

1. Kohler P, Fischer H, Schmitt J, Munhoven G. On the application and interpretation of Keeling plots in paleo climate research - deciphering delta C-13 of atmospheric CO2 measured in ice cores. *Biogeosciences* **3**, 539-556 (2006).

2. Eggleston S, Schmitt J, Bereiter B, Schneider R, Fischer H. Evolution of the stable carbon isotope composition of atmospheric CO2 over the last glacial cycle. *Paleoceanography* **31**, 434-452 (2016).

3. Bauska T*, et al.* Carbon isotopes characterize rapid changes in atmospheric carbon dioxide during the last deglaciation. *Proceedings of the National Academy of Sciences of the United States of America* **113**, 3465-3470 (2016).

4. Kohler P, Fischer H, Schmitt J. Atmospheric delta(CO2)-C-13 and its relation to pCO(2) and deep ocean delta C-13 during the late Pleistocene. *Paleoceanography* **25**, 16 (2010).

5. Keeling CD. The concentration and isotopic abundances of atmospheric carbon dioxide in rural areas. *Geochim Cosmochim Acta* **13**, 322-334 (1958).

6. Jeltsch-Thommes A, Joos F. Modeling the evolution of pulse-like perturbations in atmospheric carbon and carbon isotopes: the role of weathering-sedimentation imbalances. *Climate of the Past* **16**, 423-451 (2020).

7. Shackleton S*, et al.* Evolution of mean ocean temperature in Marine Isotope Stage 4. *Climate of the Past* **17**, 2273-2289 (2021).

8. Menviel L*, et al.* Southern Hemisphere westerlies as a driver of the early deglacial atmospheric CO2 rise. *Nat Commun* **9**, 12 (2018).

9. Menviel L, Mouchet A, Meissner KJ, Joos F, England MH. Impact of oceanic circulation changes on atmospheric delta(CO2)-C-13. *Global Biogeochemical Cycles* **29**, 1944-1961 (2015).

10. Tschumi T, Joos F, Gehlen M, Heinze C. Deep ocean ventilation, carbon isotopes, marine sedimentation and the deglacial CO2 rise. *Climate of the Past* **7**, 771-800 (2011).

11. Menviel L, Joos F, Ritz SP. Simulating atmospheric CO2, C-13 and the marine carbon cycle during the Last Glacial-Interglacial cycle: possible role for a deepening of the mean remineralization depth and an increase in the oceanic nutrient inventory. *Quat Sci Rev* **56**, 46-68 (2012).

12. Weaver AJ*, et al.* The UVic Earth System Climate Model: Model description, climatology, and applications to past, present and future climates. *Atmos-Ocean* **39**, 361-428 (2001).

13. Schmittner A, Lund DC. Early deglacial Atlantic overturning decline and its role in atmospheric CO2 rise inferred from carbon isotopes (delta C-13). *Climate of the Past* **11**, 135-152 (2015).

14. Keeling RF, Stephens BB. Antarctic sea ice and the control of Pleistocene climate instability. *Paleoceanography* **16**, 112-131 (2001).

15. Archer DE, Martin PA, Milovich J, Brovkin V, Plattner GK, Ashendel C. Model sensitivity in the effect of Antarctic sea ice and stratification on atmospheric pCO(2). *Paleoceanography* **18**, 7 (2003).

16. Archer D, Maierreimer E. Effect of deep-sea sedimentary calcite preservation on atmospheric CO2 concentration. *Nature* **367**, 260-263 (1994).

17. Peterson CD, Lisiecki LE, Stern JV. Deglacial whole-ocean delta C-13 change estimated from 480 benthic foraminiferal records. *Paleoceanography* **29**, 549-563 (2014).

18. Sigman DM, Boyle EA. Glacial/interglacial variations in atmospheric carbon dioxide. *Nature* **407**, 859-869 (2000).

19. Brovkin V, Ganopolski A, Archer D, Munhoven G. Glacial CO2 cycle as a succession of key physical and biogeochemical processes. *Climate of the Past* **8**, 251-264 (2012).

20. Kohfeld KE, Le Quere C, Harrison SP, Anderson RF. Role of marine biology in glacial-interglacial CO2 cycles. *Science* **308**, 74-78 (2005).

21. Rasmussen SO*, et al.* A stratigraphic framework for abrupt climatic changes during the Last Glacial period based on three synchronized Greenland ice-core records: refining and extending the INTIMATE event stratigraphy. *Quat Sci Rev* **106**, 14-28 (2014).

22. Veres D*, et al.* The Antarctic ice core chronology (AICC2012): an optimized multi-parameter and multi-site dating approach for the last 120 thousand years. *Climate of the Past* **9**, 1733-1748 (2013).

23. Martinez-Garcia A*, et al.* Iron Fertilization of the Subantarctic Ocean During the Last Ice Age. *Science* **343**, 1347-1350 (2014).

24. Lambert F, Bigler M, Steffensen JP, Hutterli M, Fischer H. Centennial mineral dust variability in high-resolution ice core data from Dome C, Antarctica. *Climate of the Past* **8**, 609-623 (2012).

25. Anderson RF*, et al.* Wind-Driven Upwelling in the Southern Ocean and the Deglacial Rise in Atmospheric CO2. *Science* **323**, 1443-1448 (2009).
